# Books Reviewed

**Published:** 1868-09

**Authors:** 


### Books Reviewed.

The Anatomy and Histology of the Human Eye. By A. Metz, M. D., Professor of Ophthalmology in Charity Hospital Medical College, Cleveland, Ohio. Published at the office of the Medical and Surgical Reporter, 1868.

This work on the anatomy and histology of the human eye is received by us with great pleasure, and is a text-book more urgently required than almost any other. There did not exist a treatise on this subject which included the results of the labors of the more recent observers. The author has fully accomplished his aim "to collect this material into a connected form, and in such a manner as to adapt it alike to the requirements of the medical student and of the practicing physician." The work contains seventy-five accurate engravings which illustrate the text very beautifully, adding immensely to the value of the book. It is published in a neat and tasty manner at the office of the *Medical and Surgical Reporter*, and will be received by the profession, and especially by medical students as the only text-book which embraces the results of the labors of recent observers in the anatomy and histology of the human eye. For ourselves we accept it gratefully and offer the author our hearty congratulations.

Dental Materia Medica, complete. By James W. White. Philadelphia: Samuel S. White.

This work is designed especially for dentists, and appears to us as eminently adapted to their wants. It does not treat of the nature, botanical and chemical history of the articles noticed, but is only designed to give the practical uses of the various remedies in common use by the profession, and the indications for their employment in dentistry. It seems to us that the book is invaluable to those for whom it is especially intended.

Odontalgia, commonly called Tooth-ache; its Causes, Prevention and Cure. By S. Parsons Shaw. Philadelphia: J. B. Lippincott & Co., 1868.

The object claimed by the author of this work, "is to offer a treatise which shall be so clear and precise that the physician, surgeon and dentist may alike be able to detect the symptoms of one of the most painful of all maladies, and correctly estimate the many absurd theories which prevail in regard to the various forms of facial pain."

The author endeavors to indicate the methods employed by the intelligent dentist for the prevention of tooth-ache, and points out the means to adopt, with a view of cure, so that in case of necessity, the physician or surgeon may be enabled to effect a temporary relief in a scientific manner, when a permanent cure is entrusted to the dentist. It is a book full of suggestion upon these points, and may well be in reach of physicians.

---

Lessons on Physical Diagnosis. By Alfred L. Loomis, M. D., Professor of the Institutes and Practice of Medicine in the medical department of the University of New York; Physician to Bellevue and Charity Hospitals, etc., etc. New York: Robert M. DeWitt, 1868.

The author has divided this work into fifteen lessons, the first of which is devoted to the topography of the walls of the chest, and the contents of the various regions. The second to inspection, palpation, mensuration and succussion. The third and fourth treat of percussion and auscultation, while the remaining lessons are devoted to the different diseases of the chest and abdomen. Wood cuts have been introduced, some of them, so far as we have observed, original with the author, which add greatly to the value of the work, and adapt it particularly to the wants of the medical student, rendering it more plain in its teaching than most works upon these subjects. It embraces the whole field of physical diagnosis, and is written in a direct and comprehensive manner, so that all the main and essential truths of this science are comprised within its pages. It is earnestly commended to the attention of those who would easily and thoroughly acquaint themselves with these subjects.

---

Physician's Hand-Book. New improved edition for 1869, containing all the new remedial agents. By William Elmer, M. D. Bound in English morocco, gilt edges, pocket-book form.

This new edition of the "Hand-Book" has been completely re-written and re-stereotyped throughout. Many valuable improvements and new features have been introduced, and corrections made. Price, postage free, $2.00. Price, postage free, without reading matter, $1.75.

This Pocket Hand-Book has been often brought to the attention of our readers, and the opinion expressed that few other forms were quite so well adapted to the objects in view. It is nicely bound, contains spaces for sufficient number of names, and in all respects its contents are well selected. As a pocket memorandum book it is indispensable to the physician.

